# Efficacy and Safety of Intravenous Thrombolysis on Acute Branch Atheromatous Disease: A Retrospective Case–Control Study

**DOI:** 10.3389/fneur.2020.00581

**Published:** 2020-07-07

**Authors:** Xiangbo Wu, Yang Liu, Chuang Nie, Zhiming Kang, Qunfeng Wang, Dong Sun, Huagang Li, Yumin Liu, Bin Mei

**Affiliations:** Department of Neurology, Zhongnan Hospital of Wuhan University, Wuhan University, Wuhan, China

**Keywords:** acute stroke, branch atheromatous disease, early neurological deterioration, intravenous thrombolysis, antiplatelet treatment, propensity score matching

## Abstract

**Background and Objective:** Branch atheromatous disease (BAD) is distinctive from large-artery atherosclerosis and small-vessel disease, which is single subcortical infarction caused by the occlusion of perforator's orifice. This study aimed to indicate whether intravenous thrombolysis (IVT) with alteplase could prevent early neurological deterioration (END) and improve functional outcome for patients with BAD within 4.5 h after symptom onset.

**Methods:** We retrospectively analyzed data collected from patients with BAD who were admitted to our hospital from January 2015 to August 2019. To investigate the efficacy and safety of IVT, subjects were classified into alteplase and control groups. A propensity score matching analysis was performed to control substantial heterogeneity of subgroup. The coprimary outcomes were END that is defined as an increase of ≥2 points in the National Institutes of Health Stroke Scale (NIHSS) score within 7 days after stroke, and favorable outcome at 3 months after stroke that defined by a score of 0–1 point on the modified Rankin scale (mRS).

**Results:** A total of 135 patients were eventually enrolled in this study (*n* = 51 for the alteplase group and *n* = 84 for the control group). Additionally, 42 pairs of subjects were successfully matched by propensity score matching. Intravenous alteplase within 4.5 h after stroke onset reduced the incidence of END [unadjusted odds ratio (OR), 3.32; 95% confidence interval (CI), 1.06–10.37] and improved the clinical outcome at 3 months after stroke, with more patients achieving favorable functional prognosis (mRS, 0–1 point; unadjusted OR, 0.25; 95% CI, 0.10–0.62). Patients in the alteplase group were more likely to be independent (mRS, 0–2 points) at 3 months after stroke (unadjusted OR, 0.33; 95% CI, 0.12–0.90). The rate of death or dependence (mRS, ≥4 points) in the alteplase group was also markedly lower than that in the control group (unadjusted OR, 4.06; 95% CI, 1.03–16.02).

**Conclusion:** Our findings indicated that intravenous thrombolysis may be a safe and effective therapy for patients with BAD.

## Introduction

Branch atheromatous disease (BAD) was first described as a mechanism alternative to lipohyalinosis and considered the arteriopathy closely associated with pathogenesis of single subcortical infarction (1). It is a common subtype of intracranial atherosclerotic stroke, particularly in Asian countries (2), and its prevalence was reported as high as 10.4–18.3% (3, 4). Radiographically, BAD can be misdiagnosed as lacunar infarction, while it is quite different from lacunar infarction in physiopathology. Lacunar infarction is pathologically characterized by fibrinoid degeneration or lipohyalinosis of penetrating artery (5), while BAD is caused by parent arterial disease occluding the perforator's orifice (2). Clinically, early neurological deterioration (END) was reported to frequently occur in BAD patients and often resulted in severe disability (6–8). However, to date, no optimal therapeutic approach for BAD has been presented.

Intravenous thrombolysis (IVT) with alteplase has been approved for treating acute ischemic stroke, regardless of stroke subtype (9, 10). The majority of patients with BAD was classified as minor stroke, while patients with minor stroke typically does not receive IVT. It has been previously reported that IVT neither improves clinical outcome nor prevents END in such patients, while the results of these studies could not be generalized because of their small sample size and lack of control group (11, 12). Whether IVT can effectively treat BAD has still remained elusive. In the present study, we aimed to assess whether IVT can be more effective than usual care in preventing END and improving clinical outcome for patients with BAD within 4.5 h after symptom onset.

## Materials and Methods

This is a retrospective case–control study, which was approved by the Ethics Committee of Zhongnan Hospital of Wuhan University (Wuhan, China) and was carried out in compliance with the Declaration of Helsinki. The need to informed consent was waived.

### Study Subjects

**Figure 1:**
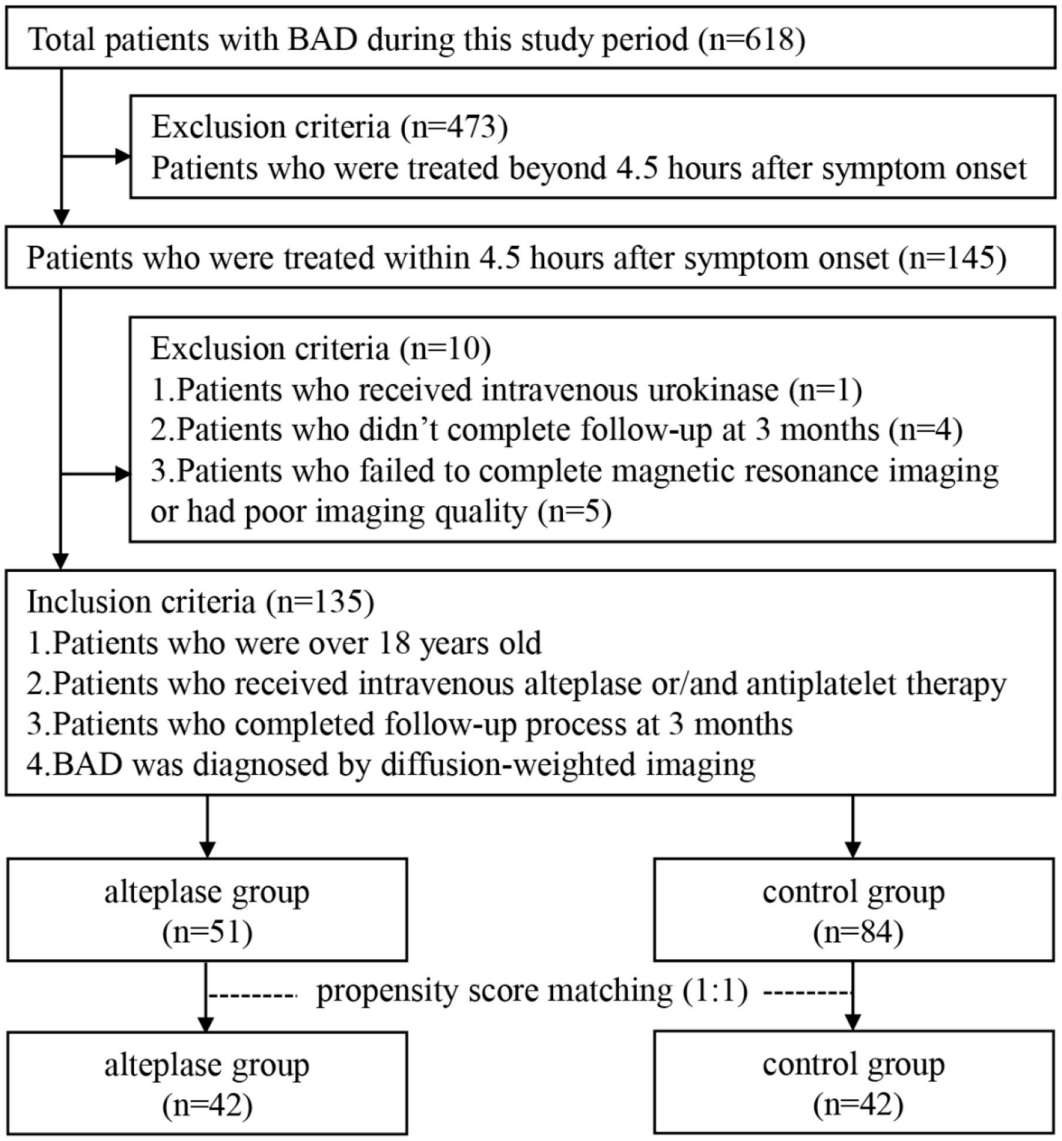
Flowchart of selection of eligible study subjects. BAD, branch atheromatous disease.

**Figure 2:**
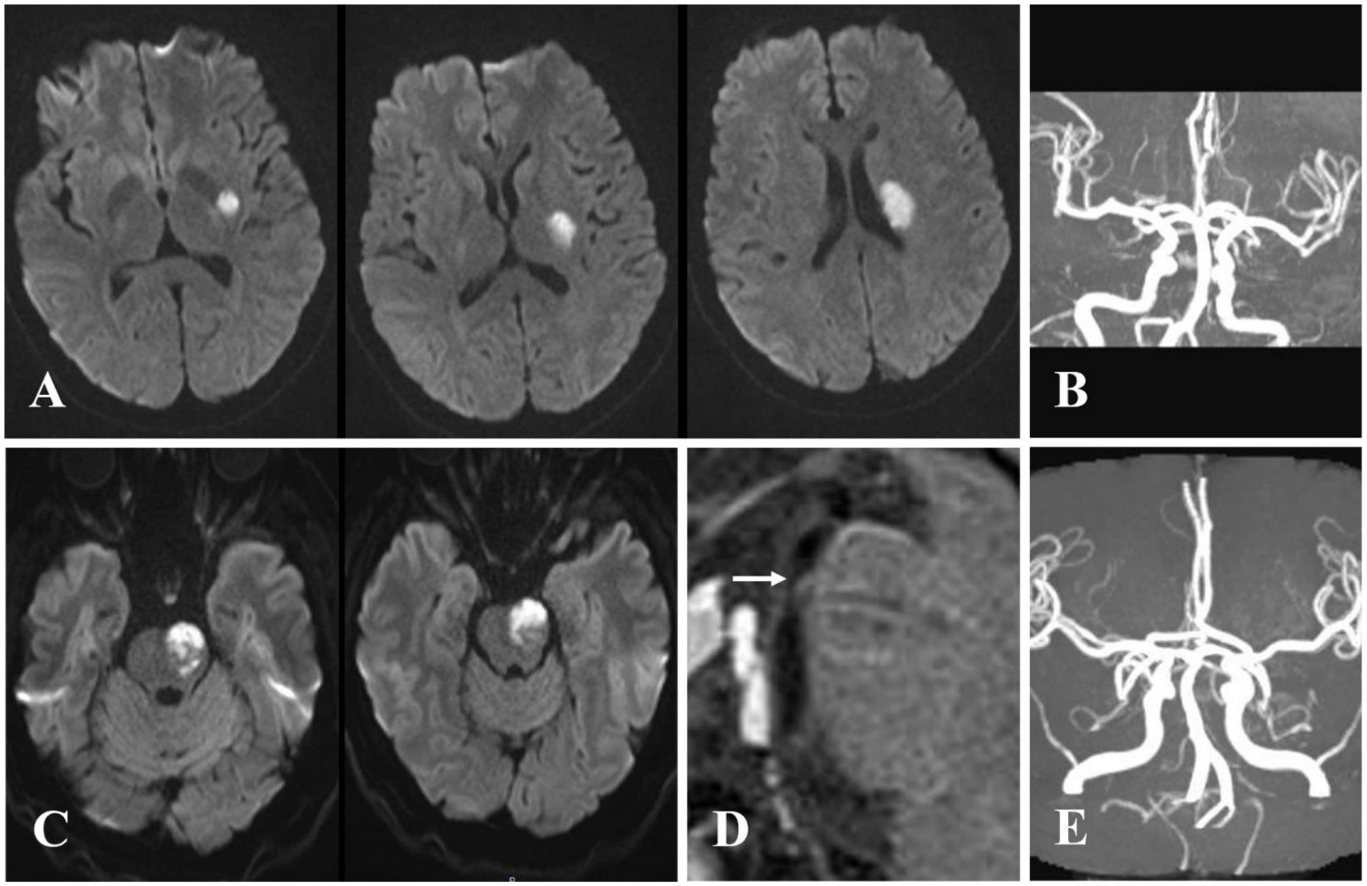
Examples of cases with branch atheromatous disease (BAD). Case 1: A 57-year-old woman with right-sided weakness. **(A)** Diffusion-weighted imaging (DWI) showing cerebral stroke in the area of lenticulostriate artery. **(B)** Magnetic resonance angiography (MRA) illustrating no significant stenosis of parent artery. Case 2: A 61-year-old man with right-sided weakness and dysarthria. **(C)** DWI showing infarction extended to the ventral pontine surface in the area of paramedian pontine artery. **(D)** Vessel wall magnetic resonance imaging (VWMRI) depicting junctional plaque in the orifice of paramedian pontine artery. **(E)** MRA displaying no significant stenosis of basilar artery.

This retrospective study was performed based on clinical data consecutively collected from patients with BAD who were admitted to Zhongnan Hospital of Wuhan University from January 2015 to August 2019. Inclusion criteria were as follows: subjects who (1) were treated within 4.5 h after symptom onset, (2) received intravenous alteplase or/and antiplatelet therapy, (3) completed follow-up process at 3 months after stroke, and (4) were diagnosed by diffusion-weighted imaging (DWI). Exclusion criteria were as follows: subjects who (1) were treated beyond 4.5 h after symptom onset, (2) received intravenous urokinase, (3) failed to complete magnetic resonance imaging (MRI) or had poor imaging quality, or (4) did not complete follow-up at 3 months after stroke. Figure 1 shows the flowchart of selection of eligible study subjects. Moreover, as illustrated in Figure 2, BAD-related infarctions were previously defined as follows (6, 11–15): (1) infarcts with a diameter ≥15 mm that involves ≥3 axial slices on DWI in the blood-supply region of lenticulostriate artery, or lesions extending to the ventral pontine surface in the blood-supply region of paramedian pontine artery; (2) neither evidence of large arterial stenosis (>50%) or occlusion, nor evidence of cardiogenic embolism.

Demographic characteristics, including age and sex, as well as clinical data, involving onset-to-needle time (ONT), baseline National Institutes of Health Stroke Scale (NIHSS) score, blood pressure at admission, baseline blood glucose levels, NIHSS score at discharge, and length of stay at hospital were recorded. Risk factors, such as hypertension, hyperlipidemia, diabetes mellitus, history of smoking, atrial fibrillation, and history of ischemic stroke were recorded as well. The clinical data were collected by two neurologists (CN and ZK).

### Therapeutic Approach

Subjects were classified into alteplase and control groups. Alteplase with a standard dose of 0.9 mg/kg was administered to patients in the alteplase group within 4.5 h after stroke onset. After confirmed no cerebral hemorrhage by non-contrast computed tomography (CT) at 24 h after intravenous alteplase, 75 mg of clopidogrel and 100 mg of aspirin were given by once daily. In the control group, 300 mg of clopidogrel and 100 mg of aspirin were initially given, followed by 75 mg of clopidogrel and 100 mg of aspirin once daily. After 21 days of undergoing dual-antiplatelet therapy, patients in the two groups were treated with aspirin or clopidogrel alone for a long time (16, 17). To avoid potential selection bias, a limited number of BAD patients who underwent mono-antiplatelet therapy were included in the current study. All patients would discontinue antiplatelet therapy if intracerebral hemorrhage was confirmed.

### Imaging Assessment

According to the American Heart Association/American Stroke Association (AHA/ASA) guideline for the early management of acute ischemic stroke (18), CT/MRI scans were performed at the time of admission prior to any treatment. The MRI of all subjects was carried out within 48 h after stroke onset, with T1WI + T2WI + DWI + T2Flair + magnetic resonance angiography (MRA) sequences, which were obtained using Siemens 3.0 T MRI scanner (Siemens AG, Munich, Germany). The BAD was diagnosed by two neurologists (XW and YL) with more than 5 years of diagnostic experience. If there was a discrepancy in their diagnoses, another senior neurologist made the final decision. The intrarater reliability of BAD was tested in 20 subjects by a single assessor with kappa (κ) coefficient equal to 0.92.

### Clinical Outcomes and Safety Variables

The modified Rankin scale (mRS) score, which ranges from 0 (no symptom) to 6 (death), was assessed at 3 months after stroke by trained neurologists (YL, CN, and ZK) who were unaware of the therapeutic measures. Besides, the severity of initial stroke was measured using NIHSS, which was conducted daily by members of our team. The coprimary outcomes were END, which was defined as an increase of ≥2 points in the NIHSS score within 7 days after stroke (3, 11) and favorable outcome at 3 months after stroke, which was defined by a score of 0–1 point on the modified Rankin Scale (19). Safety variables were death or dependence defined as the mRS score of ≥4 points at 3 months after stroke and the incidence of symptomatic intracranial hemorrhage (sICH) and parenchymal hemorrhage type 2 (PH-2) according to the European Cooperative Acute Stroke Study II (ECASS II), including hemorrhagic infarction classifications HI 1 and HI 2 and parenchymal infarction classifications PH-1 and PH-2 (20).

### Statistical Analysis

Baseline characteristics were analyzed by the Student's *t*-test, the Mann–Whitney *U*-test, chi-squared test, or Fisher's exact test. We stratified the following variables for multivariate regression analysis: the baseline NIHSS score (1–3, 4–6, or>6 points), age (≤70 or >70 years old), and onset-to-needle time (≤3 or 3–4.5 h). To minimize the effects of selection bias, propensity score matching analysis was undertaken. Matching factors included all unbalanced variables (*P* ≤ 0.1). One-to-one nearest-neighbor matching with a caliper of 0.1 was used to create two matched groups from the derived propensity score. The coprimary outcomes were the unadjusted odds ratio (OR) for END and favorable outcome (mRS score, 0–1 point), using binary logistic regression analysis to assess the odds ratio and its 95% confidence interval (CI). For safety variables, we also fitted a binary logistic regression model to evaluate the odds ratio and its 95% CI. *P* < 0.05 was considered statistically significant. Data were analyzed using SPSS 23.0 software (IBM, Armonk, NY, USA).

## Results

### Baseline Characteristics

Between January 2015 and August 2019, a total of 145 BAD patients within 4.5 h after stroke onset were consecutively enrolled in this study. According to the mentioned inclusion and exclusion criteria, 135 patients were included for final analysis (*n* = 51 for alteplase group, *n* = 84 for control group), and 10 patients were excluded (Figure 1). There was no significant di?erence in baseline characteristics and clinical outcomes between patients who were included and those who were excluded (Supplementary Table 1).

In the original cohort, patients in the alteplase group were more severely affected at admission, with a higher baseline NIHSS score (median, 7 vs. 4, *P* < 0.001), and had a lower rate of ischemic stroke history (5.9 vs. 17.9%, *P* = 0.047). Other patients' baseline characteristics showed no statistically significant difference (Table 1). A total of 42 pairs of subjects in the two groups were successfully matched by propensity score matching. All patients' baseline characteristics were comparable in our matched cohort (Table 1).

**Table 1:** The demographics and clinical characteristics of patients with branch atheromatous disease.

**Variables**	**Unmatched Cohort**			**Matched Cohort**		
	**Alteplase Group (*n* = 51)**	**Control Group (*n* = 84)**	***P*-value**	**Alteplase Group (*n* = 42)**	**Control Group (*n* = 42)**	***P*-value**
Age, mean ± SD, year	59.1 ± 12.2	64.2 ± 12.5	0.834	59.3 ± 11.9	63.7 ± 14.7	0.168
Male, *n* (%)	32 (62.7)	60 (71.4)	0.294	25 (59.5)	32 (76.2)	0.102
**Risk factors**						
Hypertension, *n* (%)	34 (66.7)	51 (60.7)	0.487	27 (64.3)	26 (61.9)	0.821
Hyperlipidemia, *n* (%)	25 (49.0)	36 (42.9)	0.485	22 (52.4)	17 (40.5)	0.274
Diabetes, *n* (%)	11 (21.6)	26 (31.0)	0.236	11 (26.2)	14 (33.3)	0.474
Smoking, *n* (%)	17 (33.3)	22 (26.2)	0.375	11 (26.2)	9 (21.4)	0.606
History of ischemic stroke, *n* (%)	3 (5.9)	15 (17.9)	0.047	3 (7.1)	5 (11.9)	0.713
**Blood pressure at admission**						
SBP, mean ± SD, mmHg	152.2 ± 21.1	154.1 ± 23.4	0.371	150 (138, 166)	155 (126, 180)	0.213
DBP, median (IQR), mmHg	88 (76, 99)	85 (76, 99)	0.414	89 (76, 95)	89 (76, 104)	0.629
Baseline blood glucose, median (IQR), mmol/L	5.4 (5.0, 6.3)	5.8 (5.1, 7.0)	0.370	5.4 (5.0, 6.3)	5.9 (5.3, 7.3)	0.181
Onset-to-needle time, median (IQR), hours	3 (3, 4)	3 (2, 4)	0.648	3 (2, 4)	3 (2, 4)	0.790
Baseline NIHSS score, median (IQR)	7 (4, 9)	4 (3, 6)	<0.001	5 (4, 8)	5 (4, 7)	0.664
NIHSS score at discharge, median (IQR)	2 (0, 5)	4 (2, 6)	0.009	2 (0, 4)	4 (3, 8)	<0.001
Hospital stay, median (IQR), days	9 (6, 12)	11 (9, 14)	0.005	9 (6, 11)	12 (9, 15)	0.001
**Infarct site**						
The lenticulostriate artery, *n* (%)	37 (72.5)	59 (70.2)	0.774	30 (71.4)	30 (71.4)	1.000
The paramedian pontine artery, *n* (%)	14 (27.5)	25 (29.8)	0.774	12 (28.6)	12 (28.6)	1.000
Dual antiplatelet treatment, *n* (%)	48 (94.1)	79 (94.0)	0.987	39 (92.9)	41 (97.6)	0.616

*SD, standard deviation; IQR, interquartile range; NIHSS, National Institutes of Health Stroke Scale; SBP, systolic blood pressure; DBP, diastolic blood pressure*.

### Outcomes in the Original Cohort

**Figure 3:**
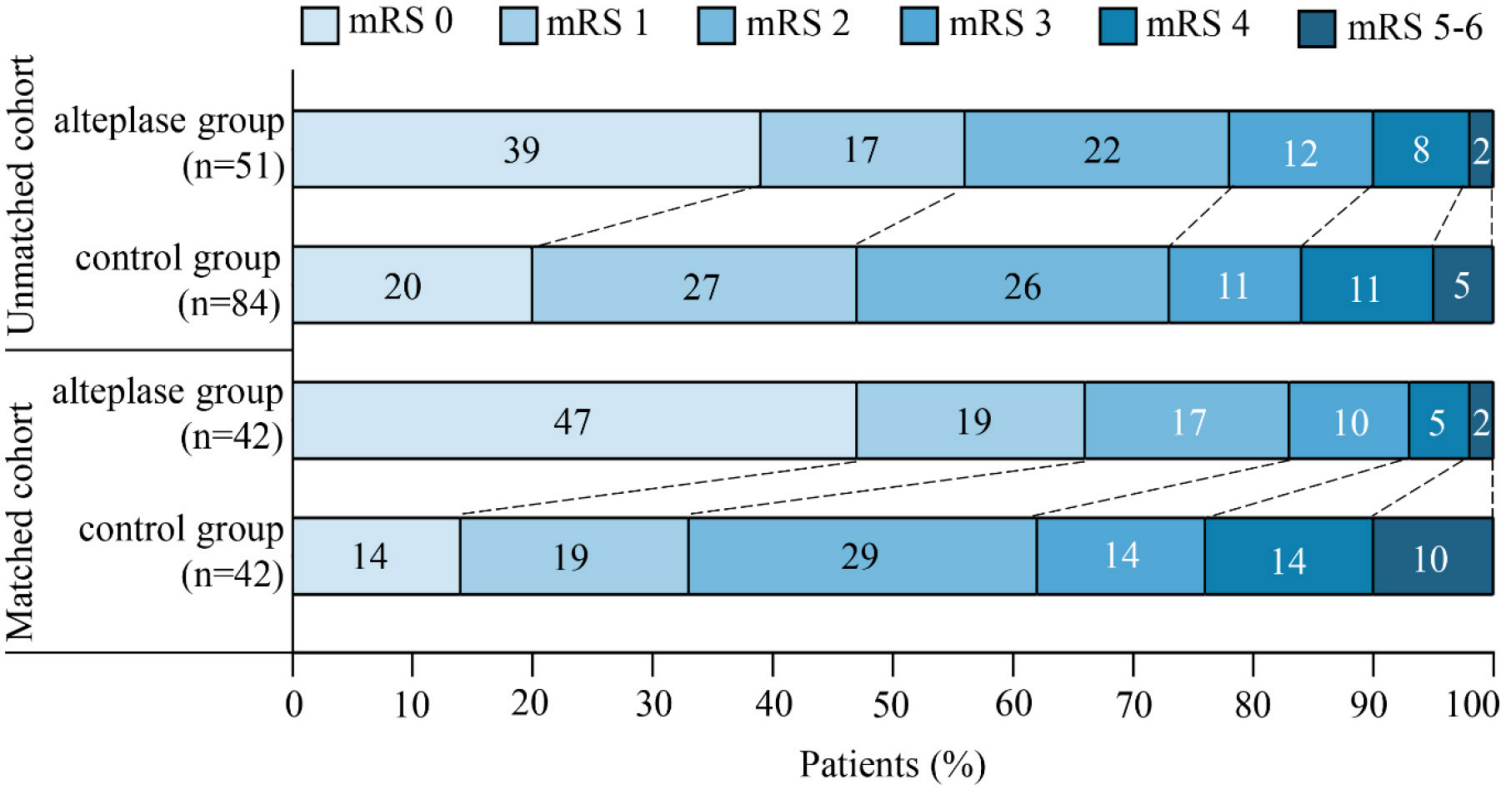
The distribution of scores on the modified Rankin scale (mRS) at 3 months after stroke was in favor of intravenous thrombolysis. The mRS scores ranged from 0 to 6, in which higher scores indicated more severe disability.

Thirty-five patients (25.9%) suffered from END in our unmatched cohort, which had longer length of stay at hospital and worse functional outcome (Supplementary Table 2). In univariate logistic regression analysis, there was significant difference in incidence of END between the two groups (unadjusted OR, 2.55; 95% CI, 1.05–6.16; *P* = 0.038). In multivariable logistic regression analysis, after adjusted for baseline NIHSS score and history of ischemic stroke, patients in the alteplase group had more favorable functional outcome [28 of 51 (54.9%) vs. 40 of 84 (47.6%); adjusted OR, 0.30; 95% CI, 0.12–0.74; *P* = 0.009]. The rate of death or dependence (mRS ≥ 4 points at 3 months after stroke) in the alteplase group was lower than that in the control group, whereas no significant difference was noted (adjusted OR, 3.14; 95% CI, 0.90–10.91; *P* = 0.072). At 3 months after stroke, a 91-year-old man in the control group died of pulmonary infection secondary to stroke, while no death happened in the alteplase group. There were two (3.9%) cases with PH-2 in the alteplase group, while no case of intracerebral hemorrhage was observed in the control group. One of the two cases had cerebral hemorrhage with no clinical symptoms, and the other had sICH in the infarcted region with severe disability (Table 2 and Figure 3).

**Table 2:** Study outcomes.

**Outcomes**	**Alteplase Group**	**Control Group**	**Binary Logistic Regression Analysis**	
**Unmatched cohort**	**(*****n*** **=** **51)**	**(*****n*** **=** **84)**	**Adjusted OR (95% CI)**	***P*****-value**
**Primary outcomes**				
mRS ≤ 1 at 3 months, *n* (%)^†^	28 (54.9)	40 (47.6)	0.30 (0.12, 0.74)	0.009
Early neurological deterioration, *n* (%)^‡^	8 (15.7)	27 (32.1)	2.55 (1.05, 6.16)	0.038
**Secondary and safety outcomes**
mRS ≤ 2 at 3 months, *n* (%)^†^	40 (78.4)	62 (73.8)	0.43 (0.16, 1.12)	0.084
mRS ≥ 4 at 3 months, *n* (%)^†^	5 (9.8)	13 (15.5)	3.14 (0.90, 10.91)	0.072
Intracranial hemorrhage, *n* (%)	2 (3.9)^*^	0	–	–
Death, *n* (%)	0	1 (2.0)	–	–
**Matched cohort**	**(*****n*** **=** **42)**	**(*****n*** **=** **42)**	**Unadjusted OR (95% CI)**	***P*****-value**
**Primary outcomes**				
mRS ≤ 1 at 3 months, *n* (%)	28 (66.7)	14 (33.3)	0.25 (0.10, 0.62)	0.003
Early neurological deterioration, *n* (%)	5 (11.9)	13 (31.0)	3.32 (1.06, 10.37)	0.039
**Secondary and safety outcomes**
mRS ≤ 2 at 3 months, *n* (%)	35 (83.3)	26 (61.9)	0.33 (0.12, 0.90)	0.031
mRS ≥ 4 at 3 months, *n* (%)	3 (7.1)	10 (23.8)	4.06 (1.03, 16.02)	0.045
Intracranial hemorrhage, *n* (%)	2 (5.0)^*^	0	–	–
Death, *n* (%)	0	1 (2.5)	–	–

*mRS, modified Rankin scale score; OR, odds ratio*.

*These models were adjusted for baseline NIHSS score and history of ischemic stroke*.

‡ *Univariate logistic regression analysis was performed due to the failure of fitting a multivariate model adjusted for baseline NIHSS score and history of ischemic stroke*.

* *There were two cases (3.9%) of parenchymal hemorrhage type 2 according to the criteria of ECASS II in the alteplase group, one of the two cases had symptomatic intracranial hemorrhage in the infarcted region and left severe disability*.

### Outcomes in the Matched Cohort

As mentioned earlier, to control substantial heterogeneity of subgroup, a propensity score matching analysis was carried out, which resulted in inclusion of 42 pairs of subjects in the matched cohort. Patients in the alteplase group remarkably improved in both primary and secondary outcomes compared with the control group. Favorable outcome (mRS, 0–1 point) at 3 months after stroke was found in 28 (66.7%) of 42 patients in the alteplase group compared with 14 (33.3%) of 42 patients in the control group (unadjusted OR, 0.25; 95% CI, 0.10–0.62; *P* = 0.003), and END was observed in 5 (11.9%) of 42 patients in the alteplase group compared with 13 (31.0%) of 42 patients in the control group (unadjusted OR, 3.32; 95% CI, 1.06–10.34; *P* = 0.039). Patients in the alteplase group were more likely to be independent (mRS, 0–2 points) at 3 months after stroke (unadjusted OR, 0.33; 95% CI, 0.12–0.90; *P* = 0.031). The rate of death or dependence (mRS, ≥4 points) in the alteplase group was also markedly lower than that in the control group (unadjusted OR, 4.06; 95% CI, 1.03–16.02; *P* = 0.045). There was one case of sICH with severe disability in the alteplase group, while one case died in the control group (Table 2 and Figure 3).

## Discussion

In 1989, Caplan initially put forward the concept of BAD and elucidated its pathomechanism, which was an occlusion or stenosis at the orifice of one or several penetrating arterial branches due to microatheroma or junctional plaque (21). However, BAD remains an underused concept in clinical practice and research in Western countries for decades (15). In contrast, BAD is a well-known disease in Asian countries, especially in Japan, China, and South Korea. Owing to routine imaging techniques that are unable to depict small vessel changes, features used to define BAD are mostly indirect, consisting of vascular territory and morphological characteristics of the ischemic lesion. The definition of BAD has not been fully set up yet, but it is universally accepted that BAD is a single subcortical infarction larger than lacunar stoke and lack of severe stenosis (≥50%) of the parent artery that supplies the regions of deep perforators, mainly the lenticulostriate arteries and pontine paramedian arteries (22). In recent years, the majority of clinical studies have followed the diagnostic criteria (12–15); however, these criteria neither reflect infarcts in the territories of other perforating arteries, nor explain the coexistence of BAD and large artery atherosclerosis. In spite of these shortcomings, no better alternative can be found based on the routine imaging techniques. Therefore, we attempted to follow the above-mentioned criteria in the present study.

The physiopathological mechanisms of BAD are complicated and have not been fully elucidated yet. Previous etiological classifications of stroke, such as Trial of Org 10172 in Acute Stroke Treatment (TOAST) (23) and Oxfordshire Community Stroke Project (OCSP) (24) are not appropriate for BAD. BAD was initially considered as a subtype of lacunar infarction by Caplan (21). However, at present, it is generally believed that BAD is distinctive from large-artery atherosclerosis and small-vessel disease. Ryoo et al. (2) demonstrated that BAD is a common and unique subtype of intracranial atherosclerotic stroke (ICAS) and has unique radiological features compared with non-BAD ICAS. Another research (22) revealed that BAD was relatively common among the embolic stroke of undetermined source (ESUS) patients and was classified as a subtype of ESUS.

In recent years, numerous scholars have demonstrated that the fluctuation or progression of neurological function is closely associated with BAD (6, 7, 22). According to different defined criteria, the prevalence of END may noticeably vary, ranging from 17 to 75% in different studies (15, 22). Neurological deterioration mainly involves motor function and often causes severe disabilities. Regrettably, a limited number of researches have concentrated on the treatment of BAD due to no international consensus about BAD classifying it as a subtype of stroke in previous etiological classifications. Although the NINDS trial demonstrated that alteplase has similar positive effect on all stroke subgroups (25), BAD treated with alteplase was found ineffective in recently conducted researches (11, 12). The current study indicated that IVT within 4.5 h after stroke onset reduced the incidence of END (unadjusted OR, 3.32; 95% CI, 1.06–10.37) and improved the clinical outcome at 3 months after stroke, with more patients achieving favorable outcome (mRS, 0–1 point; unadjusted OR, 0.25; 95% CI, 0.10–0.62), which is consistent with findings of the NINDS trial (26).

In PubMed database, only two articles concentrated on the efficacy and safety of IVT in treatment of BAD. Park et al. (11) retrospectively studied 35 patients with BAD (9 in tPA group and 26 in non-tPA group) and found that there were no statistical differences in END (*P* = 0.886) and mRS score at 3 months after stroke (*P* = 0.781) between the two groups. However, the prevalence of END in total patients (68.6%) was remarkably higher than most of BAD-related studies (12, 26–28). Subjects' poor clinical outcome may be attributed to mono-antiplatelet therapy with clopidogrel owing to higher portion of Asian individuals who are less sensitive to clopidogrel due to CYP2C19 loss-of-function alleles (29, 30). Deguchi et al. (12) assessed treatment outcomes of tissue plasminogen activator (t-PA) for hyperacute BAD within 3 h after symptom onset and demonstrated that using t-PA infusion for BAD, symptoms transiently improved, while the rate of symptom deterioration was considerable. In the present study with a medium sample size (*n* = 135), in contrast, our data unveiled that intravenous alteplase reduced the incidence of END and improved functional outcome of BAD patients. Our results may be attributable to the following reasons: first, a standard dose of alteplase (0.9 mg/kg) was given to our subjects. Whether low or standard dose of alteplase have the same efficacy on acute stroke remains controversial (31), but the majority of guidelines recommended a standard dose of alteplase for treating stroke (32). Second, dual-antiplatelet treatment was administrated to the majority of our subjects in two groups. Previous studies pointed out the efficacy of dual-antiplatelet treatment for lacunar stroke and small vessel disease. Kimura et al. (33) found that clinical progression of BAD was significantly reduced with ultra-early combination antiplatelet therapy. Berberich et al. (34) demonstrated that dual-antiplatelet therapy improved functional outcome of patients with progressive lacunar strokes.

Mortality and the incidence of sICH were insignificant in the current research. Only one patient (2%) in the alteplase group suffered from sICH according to the ECASS II criteria, which resulted in severe disability (mRS, 4 points at 3 months after stroke). Susceptibility weighted imaging (SWI) showed that this case had >10 cerebral microbleeds (CMBs) in his subcortex and brain stem. A research reported that increasing CMBs burden was closely associated with sICH after IVT treatment for acute stroke (35). Therefore, IVT is not an appropriate therapeutic approach for the mentioned patient.

There were several limitations in the current study. First, the diagnosis of BAD was carried out according to DWI data after IVT. The embolic occlusion of proximal large artery followed by alteplase-induced recanalization may come into an image that is line with BAD. Owing to the patients with cardiogenic embolism or severe arterial stenosis (≥50%) who were excluded from this study, the probability of that situation was quite low. Second, a number of variables in baseline data between the two groups were unbalanced. The propensity score matching analysis could only adjust for some confounding factors, while it could not eliminate the effect of all hidden biases. Third, this was a single-center retrospective study; the universal applicability of our results needs further study.

In conclusion,our results showed that BAD may be effectively treated with intravenous thrombolysis. However, further multicenter, prospective, randomized studies should be conducted to confirm our findings.

## Data Availability Statement

All datasets generated for this study are included in the article/Supplementary Material.

## Ethics Statement

The studies involving human participants were reviewed and approved by Medical Ethics Committee, Zhongnan Hospital of Wuhan University. The ethics committee waived the requirement of written informed consent for participation.

## Author Contributions

XW wroted and proofed the manuscript. YaL, CN, and ZK conducted follow-up and collected the data. YaL and QW conducted statistical analysis. DS, HL, and YuL revised the manuscript. BM proposed the concept and designed this study. All authors contributed to the article and approved the submitted version.

## Conflict of Interest

The authors declare that the research was conducted in the absence of any commercial or financial relationships that could be construed as a potential conflict of interest.

## Supplementary Material

Supplementary Table 1 Baseline characteristics and outcomes were compared between included and excluded groups.

Supplementary Table 2 Baseline characteristics and outcomes were compared between END and non-END groups.
