# Supplementary material for: Efficacy and Safety of Intravenous Thrombolysis on Acute Branch Atheromatous Disease: A Retrospective Case–Control Study
Source: Front Neurol. 2020 Jul 7;11:581. doi: 10.3389/fneur.2020.00581 (PMC7358343; doi:10.3389/fneur.2020.00581)
Supplement: Supplementary Table 1 — Baseline characteristics and outcomes were compared between included and excluded groups. [file Data_Sheet_1.PDF]

# 武汉大学中南医院医学伦理委员会科研项目伦理审批件
# Ethical Approval for Scientific Research Projects under Medical Ethics Committee, Zhongnan Hospital of Wuhan University

科伦 Scientific Ethical Approval[2019122]

| | | | |
|---|---|---|---|
| 项目名称<br>Project Name | 静脉溶栓治疗分支动脉粥样硬化性脑梗死的临床研究<br>Intravenous Thrombolysis for Branch Atherosclerotic Ischemic Stroke（BASIT） | | |
| 申报单位<br>Sponsor | 武汉大学中南医院 Zhongnan Hospital of Wuhan University | | |
| 科室<br>Department | 神经内科 Department of Neurology | 主要研究者<br>Primary Investigator | 梅斌 Bin Mei |
| 项目来源<br>Project Resource | 研究者发起 Researchers initiated | | |
| 审核清单<br>The List of ReviewedDocuments | 1. 伦理审查申请表 Application form for ethical review<br>2. 研究者简历 Curriculum vitae of investigator<br>3. 研究方案（V1.0,2019/9/20） Research program（V1.0,2019/9/20）<br>4. 质量管理方案 Quality management program<br>5. 风险评估 Risk assessment<br>6. 知情同意豁免申请 Apply for waiver of informed consent<br>7. 研究经费来源证明 The certificate of research funding source | | |
| 审查类型<br>review Category | 初始审查<br>Primary | 审批方式<br>Review Type | 快速审查 quick review |
| 主审委员 | 李一荣 Yirong Li 周福祥 Fuxiang Zhou | | |
| 审查意见<br>Review Opinion | 经本伦理委员会审查：批准开展该项研究。<br>According to review by Medical Ethics Committee: Approve the study.<br><br>伦理批件有效期 ：12 个月<br>Validity period: 12 months<br>主任委员或副主任委员或授权人签字：<br>Signature of Chairman or Vice Chairman：<br>武汉大学中南医院医学伦理委员会（盖章）<br>Medical Ethics Committee，<br>Zhongnan Hospital of Wuhan University（Stamp）<br>日期 Date：2019-11-4 | | |
| 注意事项 Notice： | | | |

1.请遵循 CFDA/GCP 原则和《赫尔辛基宣言》、遵循本伦理委员会批准的方案开展临床研究，保护受试者的健康与权利。Please comply with CFDA / GCP Principles and Helsinki Declaration, and carry out clinical trials according to code of ethics approved by our medical ethics committee in protection of the health and rights of human subjects

2.研究过程中，对研究方案和知情同意书等相关文件的修改，均须得到伦理委员会审查同意后方可实施。All paperwork modification related to research proposal and informed consent during the course of study are subject to approval of the Ethics Committee.

3.发生严重不良事件或影响研究风险受益比的非预期不良事件、违背方案、暂停/终止研究须及时报告本伦理委员会。Report timely to the medical ethics committee on incidents with serious adverse impact, unexpected incidents negatively affecting risk benefit ratio, breach of program or suspension / termination of study.

4.根据项目来源类别提交跟踪审查材料。 Submit follow-up review paperwork based on project resource categories

5.凡是涉及人类遗传资源出口或按照国家规定必须经有关部门审批的内容，均需在项目执行前向有关部门申报并获得批准。All contents regarding exporting of human genetic resources or approval by responsive Departments of Government, should be applied and approved in advance.

伦理委员会声明 Statement：

本伦理委员会严格按照中国 GCP 及相关法律法规组成及工作。

The Ethics Committee is well organized and works strictly in accordance with the Chinese GCP and related laws and regulations.

伦理委员会地址：湖北省武汉市武昌区东湖路 169 号门诊楼 9 楼

Address: Outpatient building, 9 floor, No.169 Donghu Road, Wuchang District, Wuhan, Hubei, China

邮编 Post Code：430071；电话 Phone：+86-027-67812787
