# Supplementary material for: Efficacy and Safety of Intravenous Thrombolysis on Acute Branch Atheromatous Disease: A Retrospective Case–Control Study
Source: Front Neurol. 2020 Jul 7;11:581. doi: 10.3389/fneur.2020.00581 (PMC7358343; doi:10.3389/fneur.2020.00581)
Supplement: Supplementary Table 2 — Baseline characteristics and outcomes were compared between END and non-END groups. [file Data_Sheet_2.PDF]

Supplementary Material

1 Supplementary Tables

TABLE 1 | Baseline characteristics and outcomes were compared between included and excluded groups.

Characteristics	Included Group (n=135)	Excluded Group (n=10)	*P* Value
Age, mean ± SD, year	62.2 ± 12.6	63.8 ± 8.2	0.222
Male, n (%)	91 (68.1)	6 (60.0)	0.728
Risk factors			
Hypertension, n (%)	85 (68.0)	7 (70.0)	1.000
Hyperlipidaemia, n (%)	61 (45.2)	5(50.0)	1.000
Diabetes, n (%)	37 (27.4)	4 (40.0)	0.469
Smoking, n (%)	39 (28.9)	2 (20.0)	0.725
History of ischemic stroke, n (%)	18 (13.3)	1 (10.0)	1.000
Blood pressure at admission			
SBP, mean ± SD, mmHg	153.4 ± 22.5	152.0 ± 19.1	0.551
DBP, median (IQR), mmHg	85 (76, 99)	79 (73, 95)	0.525
Baseline blood glucose, median (IQR), mmol/L	5.6 (5.0, 7.0)	6.1 (5.4, 9.8)	0.224
Onset-to-needle time, median (IQR), hours	3 (2, 4)	3 (3, 4)	0.620
Baseline NIHSS score, median (IQR)	5 (3,7)	5 (4, 7)	0.844
NIHSS score at discharge, median (IQR)	3 (2, 6)	4 (2, 5)	0.726
Hospital stay, median (IQR), days	10 (8, 13)	11 (9, 13)	0.879
Infarct site			
The lenticulostriate artery, n (%)	96 (71.1)	8 (80.0)	0.725
The paramedian pontine artery, n (%)	39 (28.9)	2 (20.0)	0.725
Intravenous thrombolysis, n (%)	51 (37.8)	3 (30.0)	0.744
Dual-antipltelet treatment, n (%)	129 (95.6)	9 (90.0)	0.400
Early neurological deterioration, n (%)‡	35 (25.9)	3 (30.0)	0.722
mRS scores at 3 months， median, IQR	1 (0, 2)	2 (0, 3)	0.746

Abbreviations: SD, standard deviation; IQR, interquartile range; NIHSS, National Institutes of Health Stroke Scale; mRS, Modified Rankin Scale score; SBP, systolic blood pressure; DBP, diastolic blood pressure.

TABLE 2 | Baseline characteristics and outcomes were compared between END and non-END groups.

**Characteristics**	**END Group (n=35）**	**Non-END Group (n=100)**	***P* Value**
Age, median, IQR, year	62 (56, 67)	62 (52, 72)	0.918
Male, n (%)	24 (68.8)	68 (68.0)	0.950
Risk factors			
Hypertension, n (%)	22 (62.9)	63 (63.0)	0.988
Hyperlipidaemia, n (%)	19 (54.3)	42(42.0)	0.209
Diabetes, n (%)	12 (34.3)	25(25.0)	0.289
Smoking, n (%)	7 (20.0)	32 (32.0)	0.178
History of ischemic stroke, n (%)	6 (17.1)	12 (12.0)	0.441
Blood pressure at admission			
SBP, mean ± SD, mmHg	158.7 ± 18.3	151.6 ± 23.7	0.223
DBP, median (IQR), mmHg	85 (78, 99)	85 (75, 96)	0.460
Baseline blood glucose, median (IQR), mmol/L	5.9 (5.1, 7.5)	5.6 (5.0, 6.8)	0.255
Onset-to-needle time, median (IQR), hours	4 (2, 4)	3 (2, 4)	0.549
Baseline NIHSS score, median (IQR)	5 (3,6)	5 (3, 8)	0.369
NIHSS score at discharge, median (IQR)	6 (3, 8)	3 (1, 4)	＜0.001
Hospital stay, median (IQR), days	12 (9, 15)	10 (8, 12)	0.018
Infarct site			
The lenticulostriate artery, n (%)	26 (74.3)	70 (70.0)	0.630
The paramedian pontine artery, n (%)	9 (25.7)	30 (30.0)	0.630
Intravenous thrombolysis, n (%)	8 (22.9)	43(43.0)	0.034
Dual-antipltelet treatment, n (%)	32 (91.4)	95 (95.0)	0.427
mRS scores at 3 months，median, IQR	2 (2, 4)	1 (0, 2)	＜0.001

Abbreviations: SD, standard deviation; IQR, interquartile range; NIHSS, National Institutes of Health Stroke Scale; mRS, Modified Rankin Scale score；END, early neurological deterioration; SBP, systolic blood pressure; DBP, diastolic blood pressure.
